# Experimental and Numerical Study of Fracture Behavior of Rock-Like Material Specimens with Single Pre-Set Joint under Dynamic Loading

**DOI:** 10.3390/ma14102690

**Published:** 2021-05-20

**Authors:** Bo Pan, Xuguang Wang, Zhenyang Xu, Lianjun Guo, Xuesong Wang

**Affiliations:** 1 School of Civil and Resource Engineering, University of Science and Technology Beijing, Beijing 100083, China; bopan07@foxmail.com (B.P.); lruoxi008@sina.com (X.W.); 2 School of Mining Engineering, University of Science and Technology Liaoning, Anshan 114051, China; 3 School of Architecture and Civil Engineering, Shenyang University of Technology, Shenyang 110870, China; wshellson@foxmail.com

**Keywords:** joint angle, rock-like material, SHPB, microscopic damage, stress field characteristics

## Abstract

The Split Hopkinson Pressure Bar (SHPB) is an apparatus for testing the dynamic stress-strain response of the cement mortar specimen with pre-set joints at different angles to explore the influence of joint attitudes of underground rock engineering on the failure characteristics of rock mass structure. The nuclear magnetic resonance (NMR) has also been used to measure the pore distribution and internal cracks of the specimen before and after the testing. In combination with numerical analysis, the paper systematically discusses the influence of joint angles on the failure mode of rock-like materials from three aspects of energy dissipation, microscopic damage, and stress field characteristics. The result indicates that the impact energy structure of the SHPB is greatly affected by the pre-set joint angle of the specimen. With the joint angle increasing, the proportion of reflected energy moves in fluctuation, while the ratio of transmitted energy to dissipated energy varies from one to the other. NMR analysis reveals the structural variation of the pores in those cement specimens before and after the impact. Crack propagation direction is correlated with pre-set joint angles of the specimens. With the increase of the pre-set joint angles, the crack initiation angle decreases gradually. When the joint angles are around 30°–75°, the specimens develop obvious cracks. The crushing process of the specimens is simulated by LS-DYNA software. It is concluded that the stresses at the crack initiation time are concentrated between 20 and 40 MPa. The instantaneous stress curve first increases and then decreases with crack propagation, peaking at different times under various joint angles; but most of them occur when the crack penetration ratio reaches 80–90%. With the increment of joint angles in specimens through the simulation software, the changing trend of peak stress is consistent with the test results.

## 1. Introduction

**Figure 1:**
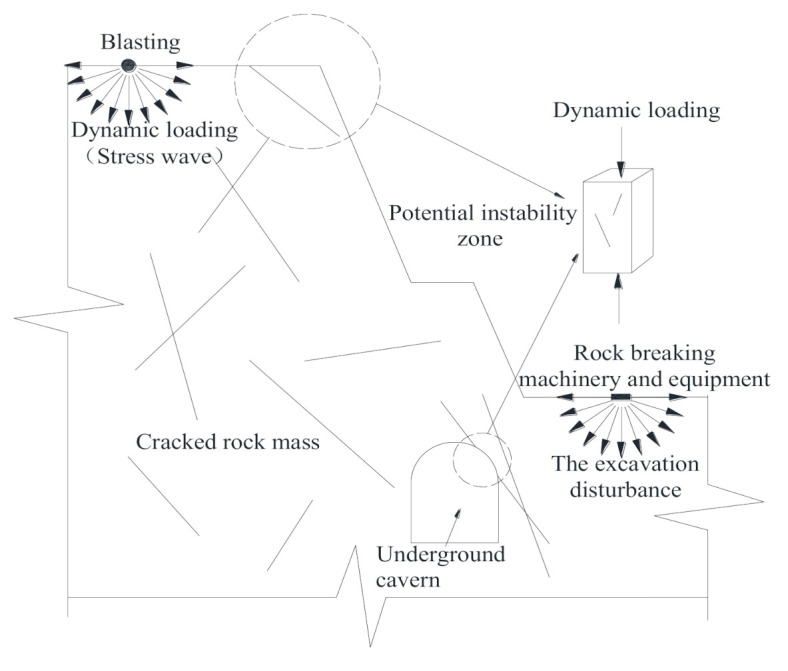
Schematic diagram of instability zone of cracked rock mass.

Rock masses in nature are composed of various discontinuities, such as faults, joints, fissures, etc. These defects that determine the mechanical behaviors of rock masses tend to develop gradually with the change of the external environment. The crack propagation under the influence of excavation disturbance and the slope shedding under the influence of explosion are all macroscopic failure phenomena manifested due to crack initiation, propagation, and coalescence in rock mass under the load effect (as shown in Figure 1). Therefore, it is very important have an accurate understanding of the deformation and failure characteristics of rock mass with discontinuities to ensure the stability and the construction economics of rock mass [1,2,3].

As one of the most common discontinuities in rock masses, joints have always been the research focus of experts and scholars in the field of rock mechanics. In the 1970s, Jaeger J C [4] adopted the Mohr–Coulomb criterion to analyze the strength of rock mass with joints and took the lead in elaborating on the single discontinuity theory. As one of the active test methods of dynamic load, SHPB has been widely used to impose a dynamic load on a material specimen akin to that which the material will experience in dynamic situations. Through changing the loading strain rate, the dynamic properties of jointed rock specimen were analyzed systematically [5]. For further enrichment, the research programs on rock joints have been improved through the change of the parameters of the joint fillings [6], coupling the static and dynamic load [7], and pretreatment under freezing and thawing conditions [8,9], etc. With continuous improvement of finite element and discrete element software, numerical simulation tests of the dynamic fracture process of joint specimens have been carried out under the same impact pressure, so as to replenish the crack propagation law of specimens [10,11,12,13]. Case exploration with various numerical simulation software [14,15], will provide a new idea for the research on the damage evolution of the jointed rock mass. After analysis of the deformation and failure of concentrated parallel jointed rock mass with different spacing and number [16], as well as the failure crack propagation process [17,18], disparities can be seen in the crack initiation location of different types of crack propagation. Besides, SHPB test results can be verified and further analyzed through numerical simulation (ANSYS) [19]. With the introduction of nuclear magnetic resonance (NMR) technology, the pace of microscopic research on rock damage has been accelerated. To be specific, the NMR technology has been used to clarify the change process of damage and fracture of specimens [20], as well as the structural change of specimens by detecting NMR transverse relaxation time T_2_ distribution and porosity change under different prestressing effects [21] with the starting point of rock failure [22] studied too.

The present work analyzes the impact of dynamic loading on the penetration of jointed rock specimen with preset multiple angles, extracts the surface crack of specimen, obtains inner pore distribution of the specimen by means of nuclear magnetic resonance (NMR) imaging technology, and analyzes how different crack angles affect the crack propagation mode of specimens under impact load. Meanwhile, with help of numerical simulation, the work analyzes the crack propagation process and the change of stress zone to provide reference for solving engineering problems of the jointed rock mass.

## 2. Materials and Methods

### 2.1. Specimen Preparation

P.O 42.5 ordinary Portland cement is used in the test, and the particle size of fine sand and aggregate shall not be larger than 0.6 mm. The cement, water, and fine sand are proportioned as 1:0.5:2, according to weight. After even stirring, the mixture was injected into the mold and subject to the vibration for air exhaust. The demolding is then carried out after solidification and hardening for 48 h, and the cement mortar specimens are cured at room temperature (20 °C) for 28 days in saturated Ca(OH)_2_ aqueous solution. For better analyzing the failure mode of the specimens and revealing the crack initiation and development, the length-diameter ratio of the specimen is adjusted from the general range of 0.4~0.6 [23] to 2.0~2.2 on the basis of specimen sizes in the quasi-static compression test.

**Figure 2:**
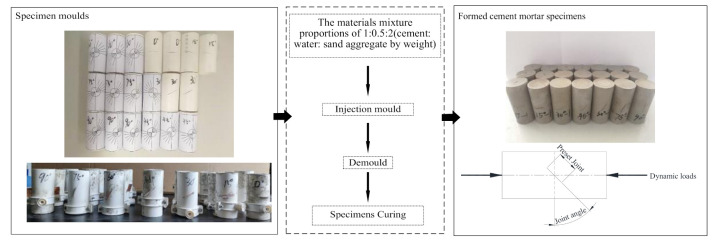
Specimen preparation process.

The size of joint angle specimens is Φ46 mm × 100 mm, the angles adopt 0°, 15°, 30°, 45°, 60°, 75°, 90°, separately, the joint length is 20 mm, and the joint thickness is 0.2 mm. Partial cutthrough is conducted along the radial direction (full cutthrough in front of and behind the joint, and non-full cutthrough around the joint. The preparation process of specimens is shown in Figure 2). The joints are located in the middle of the specimens, with 3 specimens for each angle, 21 specimens in total. The design size and specification of non-jointed specimens are the same as above, totally 12 in number.

In the process of specimen preparation, the copper sheet with a thickness of 0.2 mm is placed along the opening gap of the mold and gently shaken out during demolding. In order to prevent the copper sheet from bonding with cement mortar too closely, mineral oil is evenly coated on both sides of the copper sheet. The length of the mold is increased to 110 mm during the mold production. After demolding, the specimen is cut to meet the ISRM requirements.

### 2.2. Test Program

To eliminate data deviation caused by the specimen production as much as possible, the initial damage analysis is carried out on the cement mortar specimens of each group. Firstly, the specimens are saturated in water by ZYB-II Vacuum device. Then the initial porosity of the specimens is measured with MacroMR12-150H-I (Suzhou Niumag Analytical Instrument Corporation, Suzhou, China) nuclear magnetic resonance imaging analyzer to eliminate the specimens with prominent differences. To define the strength of impact pressure and provide reliable data reference for continuous studies, a group of non-jointed cement mortar specimens (B-1~B-3) are subject to the quasi-static uniaxial compression test, according to the operation points of the test instrument. The physical mechanics parameters of the cement mortar specimens are as shown in Table 1.

The impact pressure determines the crushing degree of the specimen. Specimens should be relatively complete to study the crack propagation law of jointed rock masses with different geometric characteristics. Additionally, since the porosity test and imaging with nuclear magnetic resonance technology also require the tested objects to maintain in good shape, and too many fragments may lead to errors in porosity measurement results and add to test difficulty, it is necessary to find an appropriate pressure value, control the incident energy within a certain range to ensure that the crushing effect of the specimen can meet the requirements of subsequent tests and analysis.

**Figure 3:**
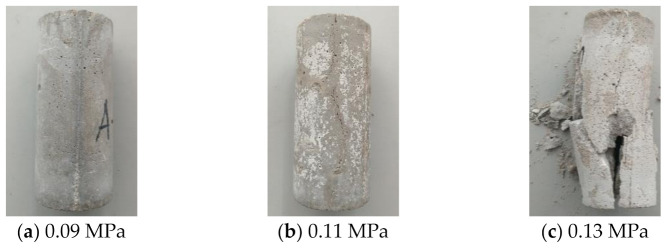
The specimens’ fragmentation under different impact pressure.

**Figure 4:**
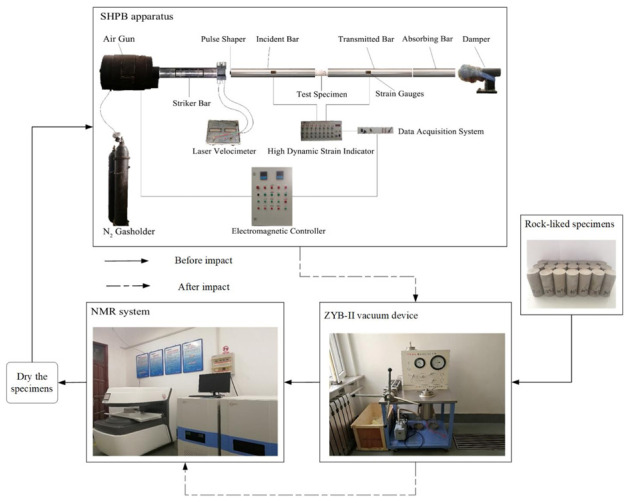
Schematic diagram of the experimental procedures.

According to the quasi-static test results, the dynamic strength test is carried out on three specimens without joints, namely A-1, A-2, and A-3, to find the cylinder pressure with critical dynamic failure strength, and SHPB impact test is carried out with this critical pressure value. Figure 3 shows the broken state of specimens without joints in group A under different impact pressures. In Figure 3a, under the impact pressure of 0.09 MPa (the velocity of the impact warhead is about 6.22 m/s), there are slight damages near the upper end face of the specimen, and there is no obvious damage feature on the whole, which does not meet the test requirements; In Figure 3b, under the impact pressure of 0.11 MPa (the velocity of the impact warhead is about 7.46 m/s), obvious cracks appear on both end faces and sides of the specimen, which meets the conditions of the test on the law of crack development and propagation. The whole specimen is not too broken and meets the requirements of nuclear magnetic resonance test. In Figure 3c, under the impact pressure of 0.13 MPa (the velocity of the impact warhead is about 8.97 m/s), there are extensive damages on the specimen, and too many fragments and powders, which is not conducive to the implementation of nuclear magnetic resonance test. Therefore, the impact pressure of SHPB test is finally determined to be 0.11 MPa. See Table 2 for the specific scheme, and the test process is shown in Figure 4.

## 3. Results and Discussion

### 3.1. Influence of Joint Angle on Energy Dissipation

#### 3.1.1. Energy Density per Unit Time

The energy input of the Split Hopkinson Pressure Bar (SHPB) device is realized through the impact of the impingement warhead. According to the one-dimensional stress hypothesis, the energy attenuation caused by stress wave propagation in the bar is ignored, and the energy of the incident wave *E*_I_, the reflected wave *E*_R_, and the transmitted wave *E*_T_ can be expressed as below [24]:

(1) $$E_{I}=A_{0}c_{0}E_{0}\int_{0}^{t}\varepsilon_{i}^{2}(t)dt$$

(2) $$E_{R}=A_{0}c_{0}E_{0}\int_{0}^{t}\varepsilon_{r}^{2}(t)dt$$

(3) $$E_{T}=A_{0}c_{0}E_{0}\int_{0}^{t}\varepsilon_{t}^{2}(t)dt$$

where *A*_0_ and *E*_0_ are the cross-sectional area and Young’s modulus of the bar, and *c*_0_ is the one-dimensional longitudinal stress wave velocity of the bar.

In the process of the impact test, the energy wasted by the contact interface between the pressure bar and the specimen is generally ignored. It is considered that the energy of incident wave *E*_I_ is all converted into the reflected wave energy *E*_R_, the transmitted wave energy *E*_T_, and the dissipated specimen energy *E*_D_.

(4) $$E_{I}=E_{R}+E_{T}+E_{D}$$

To eliminate the influence of specimen size on the specimen energy dissipation, the dissipated energy by unit volume is used to characterize the energy of stress wave absorbed by specimen [25] and can be expressed as Equation (5). However, in addition to the specimen size, the duration of the stress wave acting on the specimen also affects the energy dissipation of the specimen. The energy dissipation per unit volume in defined unit time is used as a new index to evaluate energy dissipation of the specimen, referred to as energy density per unit time, as shown in Equation (6). Since the starting and ending time of the reflected wave and the transmitted wave are basically the same, the elapsed time of the reflected wave can be taken as the action time of the stress wave in the specimen

(5) $$E_{V}=E_{D}/V_{S}$$

(6) $$E_{\mathrm{VT}}=E_{D}/(V_{S}T_{R})$$

where *E*_V_ is the dissipated energy per unit volume, *E*_VT_ is energy density per unite time, *V*_S_ is the volume of the specimen, *T*_R_ is the elapsed time of the reflected wave.

#### 3.1.2. Analysis of Energy Dissipation

**Figure 5:**
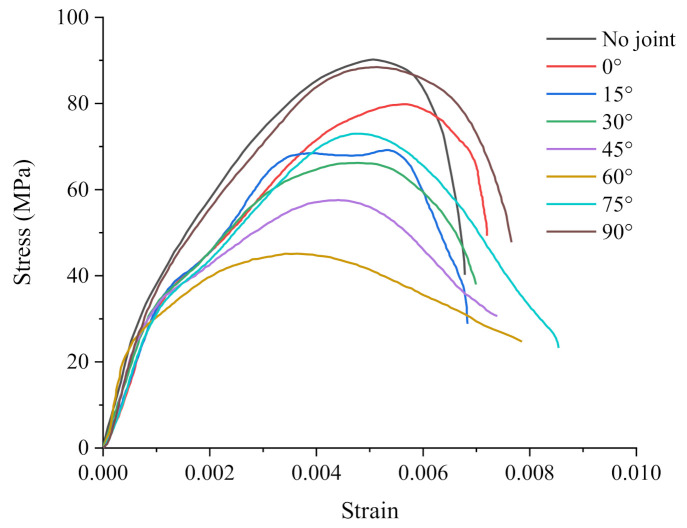
Stress–strain curves of specimens with joint angles.

Figure 5 shows that that the stress–strain curve of the specimen shows different changes due to different pre-set joint angles after the peak stress point. The intact one, with 0° and 90° joint angle, shows brittle failure characteristics, namely, when the external dynamic load is larger than the peak resistance of the specimen, the intact one rapidly fails and the energy is released rapidly. For specimens with 45°, 60°, and 75° joint angles, there is an obvious inflection point at the boundary between elasticity and compaction at the beginning of the curve. After reaching the peak stress, it shows a gentle downward trend. The post-peak change rates vary with different joint angles. Specifically, the stress–strain curves of the specimens with 45° and 75° joint angles show good symmetry. However, the peak strength of the specimens with 45° joint angle is far less than specimens with 75° joint angle, meaning that the specimen is prone to damage with 45° internal joint angle, with the absorption and consumption of external energy relatively stable, accompanied by a certain residual strength. For the specimen with 60° joint angle, the curve shows the gentlest change after the peak point, because the specimen slides along the joint surface after compaction, thus the strain change intensifies without the influence of excessive stress. For specimens with 15° joint angle, the curve has two peaks, and the stress resistance decreases after the first peak point, and then rebounds. The reason is that in the process of the test, the bar is subject to the secondary contact, which accelerates the softening process of the specimen, thus showing similar brittle failure characteristics.

**Figure 6:**
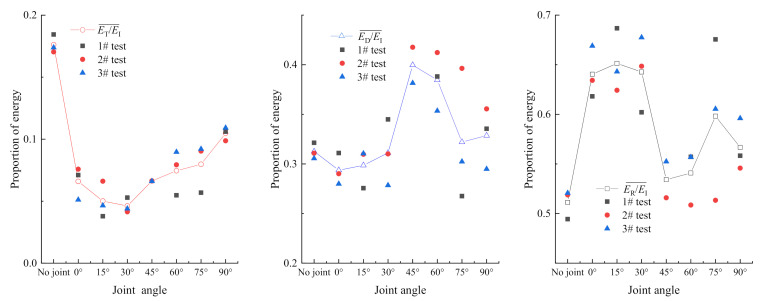
Proportion of energy with joint angles.

Waveform data are processed with Equations (1)–(4) to obtain the incident energy *E*_I_, reflected energy *E*_R_, transmitted energy *E*_T_, and dissipated energy *E*_D_ of the specimen. Due to human manipulation and other inevitable factors, the incident energy cannot be completely consistent even with 0.11 MPa impact pressure adopted in the test. To reduce the influence of incident energy on test analysis, the ratio method [26,27] is used to analyze the energy structure of specimens with different joint angles. Figure 6 shows the ratio curve of energy reflection, energy transmission, and energy dissipation as the crack angle increases. As per the analysis, the ratio of energy reflection to incident energy, *E*_R_/*E*_I_, varies with the joint angles as follows: the proportion of energy reflection increases for specimens with no joint to 0° joint angle; no noticeable change has been observed in the proportion of energy reflection of the specimen with 0°–30° joint angle, which maintains in the range of 64–65%; the proportion of energy reflection of the specimen with 30° joint angle reaches the inflection point and then declines, and the proportion of energy reflection of specimens with 45°–60° joint angles touches the lowest point of about 53%; the proportion of energy reflection of the specimen with 60°–90° joint angles increases slightly.

The ratio of energy transmission to incident energy, *E*_T_/*E*_I_, varies with joint angles as follows: the proportion of energy transmission of specimens decreases gradually, the decline slowing down gradually from no joint to 30° joint angle; the proportion of energy transmission of specimens with joint angles of 30°–90° increases gradually. The ratio of energy dissipation to incident energy, *E*_D_/*E*_I_, varies with joint angles as follows: the proportion of energy dissipation of specimens increases gradually from no joint to 45° joint angle, with slow increase from 0° to 30°and fast increase from 30° to 45°; the energy dissipation of specimens with joint angles of 45°–90° decreases gradually. When the joint angle falls in the range of 45°–60°, the energy dissipation of the specimen reaches the highest level, indicating that these specimens can make full use of the incident energy, and are conducive to the improvement of the crushing effect.

Generally speaking, with small differences in incident energy, the crack angle has significant impact on energy reflection, energy transmission, and energy dissipation, and there is a strong correlation between energy reflection and energy dissipation. The relation between the average ratio and the ratio of each specimen is shown in Figure 6.

#### 3.1.3. Analysis of Energy Density per Unit Time

**Figure 7:**
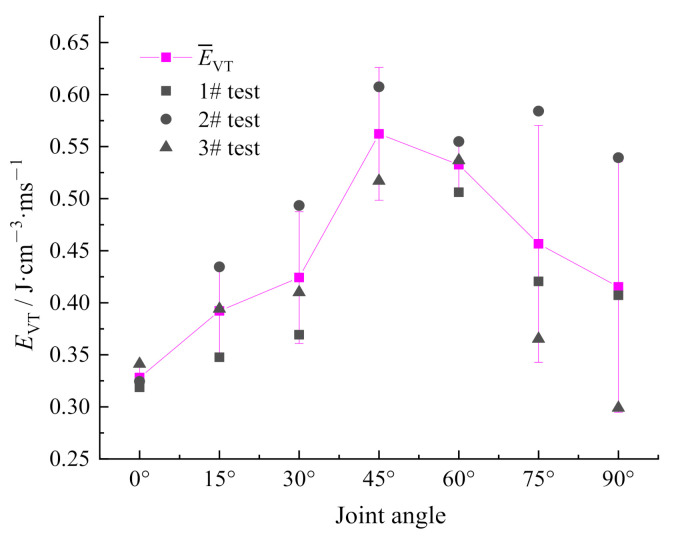
Relationship between energy density per unit time and joint angles.

Based on the introduction of the energy density model above, the elapsed time of reflected wave of specimens with the joint angle of 0°–90° is extracted. Combined with the energy parameters in Table 3, Equation (6) is used to calculate the energy density per unit time *E*_VT_ of specimens with different joint angles, as shown in Figure 7. It can be seen that the energy density per unit time *E*_VT_ of specimens with the joint angle of 0°–90° increases at first and then decreases as the angle increases. When the joint angle falls in the range from 0° to 45°, the *E*_VT_ value increases by 71.3%. Meanwhile, within this range, the energy absorbed by specimens increases continuously. The energy absorption of the specimen with 45° joint angle reaches the highest value 0.5623 J·cm^−3^·ms^−1^. When the joint angle falls in the range from 45° to 90°, the *E*_VT_ value decreases by 26.2% even reaches the lowest level, and the energy absorbed by the specimen gradually decreases within this range. The *E*_VT_ value maintains at a higher level when the joint angle falls in the range from 45° to 60°, indicating that the jointed specimens within this range could better absorb incident energy, and the more crack initiation and propagation within the specimen consume the more energy under impact consumed.

### 3.2. Analysis on Crack Propagation of Specimens with Different Joint Angles

**Figure 8:**
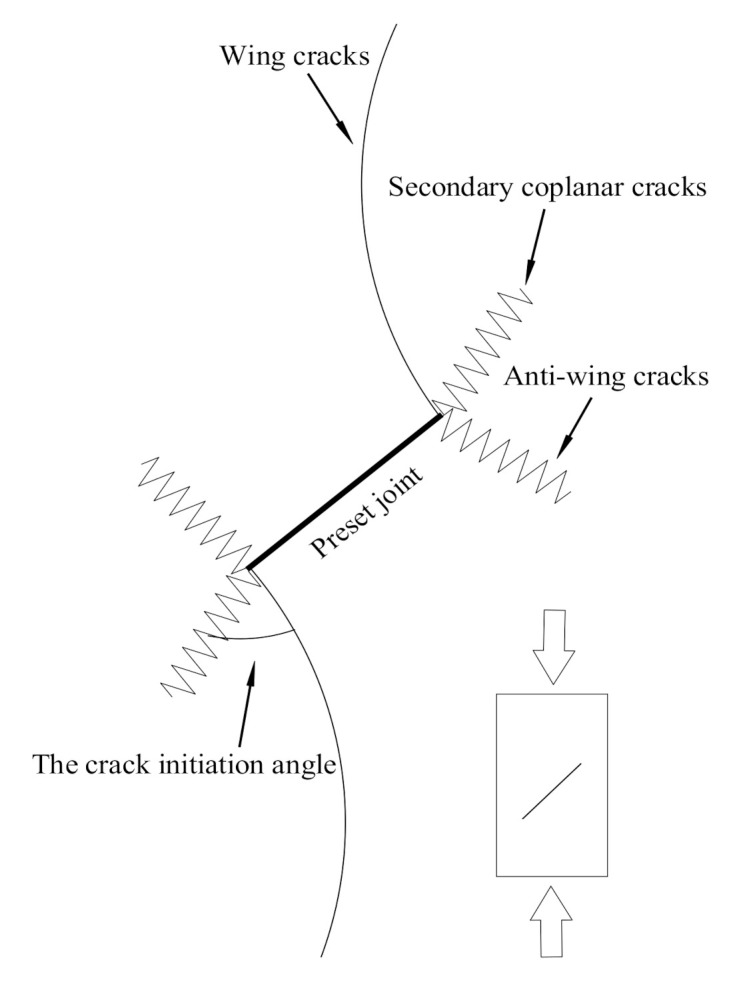
A schematic diagram of crack propagation of jointed rock mass.

Under the action of external load, the joint end of rock masses containing original joints is vulnerable to damage, which can be divided into tensile failure and shear failure according to stress types. The main form of tensile failure is wing crack, and that of shear failure is anti-wing crack and secondary coplanar crack. The wing crack usually initiates at the joint end and develops gradually parallel to the loading direction. The crack direction of the anti-wing crack initiates and develops opposite to that of the wing crack. Secondary coplanar cracks also originate at the joint end and develop coplanar with the joint. The crack propagation mode of the jointed rock mass is shown in Figure 8 [28].

**Figure 9:**
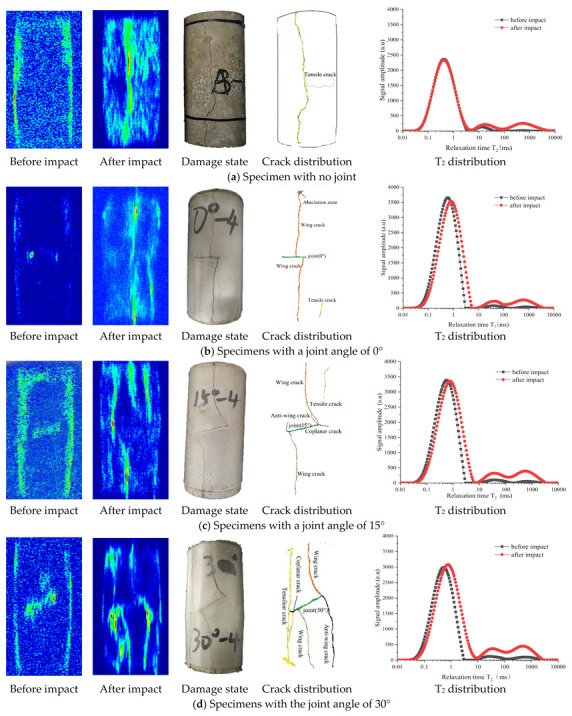

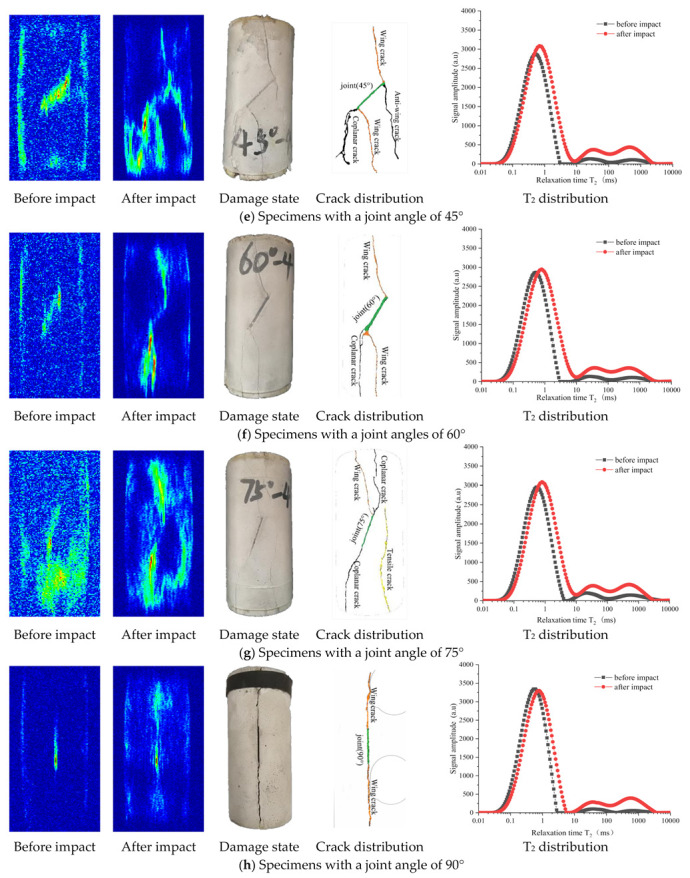
MRI effect, crack morphology, and T_2_ distribution of specimen with different joint angles.

Figure 9 shows the nuclear magnetic resonance imaging and surface crack distribution of specimens with different joint angles. It can be seen in the first two pictures in (a) that the hot spots increase after impact, and there is an obvious strip area with continuous hot spots, and the rest of the hot spots are randomly distributed on both ends of specimen, which indicates that the internal damage of the specimen after impact load increases significantly, and a through crack forms. On the whole, the damage on both ends of the specimen is higher than that in the middle part. It can be seen from the last three pictures that the complete specimen without joints produces radial tensile stress under the action of axial compressive stress, and a top-to-bottom tensile crack on the specimen is generated in the middle part, which is almost parallel to the direction of axial stress and belongs to brittle splitting failure.

It can be found that in (b), there are increased scattered hot spots at both ends of the specimen with the joint angle of 0° and both ends of the joint after the impact, and three continuous axial strip hot spots near the center and on the lower right side of the specimen, indicating that the number of small pores at both ends of the specimen and joint increases but those pores are not connected as cracks. Three cracks are formed at the center and lower right of the specimen. Under the influence of dynamic load, the specimen is subject to the tensile stress in the radial direction, a wing crack is initiated at the center of the upper part of the preset joint and connected with the upper end of the specimen. Another wing crack is initiated at the right end of the preset joint and connected with the lower end of the specimen, forming a tensile crack at the lower right part of the specimen. The initiation angle of the two wing cracks is about 90°.

After the specimen with the preset 15° joint angle is subject to the impact, there are four continuous axial strip hot spots in the specimen. Two wing cracks develop at both ends of the preset joint, with one anti-wing crack developing on the left side and a short secondary coplanar crack on the right side. A tensile crack is formed on the upper right of the specimen, and the crack initiation angle of the two wing cracks increases to 105° compared with that of the specimen with the joint angle of 0°.

After the specimen with preset 30° joint angle is subject to impact, the macroscopic cracks are well developed. A short secondary coplanar crack develops on the left side, an anti-wing crack on the right side, and a tensile crack connecting up and down is formed on the left side of the specimen. The crack initiation angle of the two wing cracks is about 95°, slightly larger than that of the specimen with the joint angle of 0°.

The strip area with continuous hot spots area of the specimen with the joint angle of 45° is mainly concentrated in the lower part of the specimen, indicating more serious damage in the lower part of the specimen. A number of cracks are developed, with wing cracks developed at both ends of the specimen joints. The crack initiation angle of the wing crack is about 86°, which is close to the angulation of the specimen with the joint angle of 0°. An anti-wing crack develops at the right end of the joint, and a secondary coplanar crack at the left.

After the impact action, there are continuous strip areas of hot spots on the upper and lower parts of the specimen with the joint angle of 60°, and hot spot areas in the lower part are fan-shaped, that is, a crack is formed on the upper part of the specimen, and a fan-shaped failure area is formed on the lower part of the specimen. Wing cracks are developed at both ends of the specimen joints, a secondary coplanar crack on the left side of the joint. The crack initiation angle of the two wing cracks decreases to about 74°.

There are three strip continuous hot spots near the center and the lower right side of the specimen with the joint angle of 75°, and two hot spots near the center are fan-shaped. There are secondary coplanar cracks at both ends of the joint, and a wing crack at the right end of the joint. The crack initiation angle of the wing crack continues to decrease to about 61°.

After impact, there is only one continuous strip area of hot spot from top to bottom of the specimen with the joint angle of 90°, indicating that only one through crack is formed inside the specimen and only one coplanar wing crack is developed, as shown in Figure 9h.

According to the magnetic resonance imaging results of specimens after impact, specimens with joint angles of 15°, 30°, and 45° have significantly more strip continuous areas of hot spots than those with joint angles of 0°, 60°, 75°, and 90°, indicating that the former possess more macroscopic cracks than the latter. From the point of view of the crack development state of specimens, the specimens with no joint angle, 0°, and 90° joint angle are mainly affected by tensile stress to produce tensile failure or splitting failure. The wing crack of the specimen with 0° joint angle originates in the middle part of the joint, and the tensile crack develops on the specimen with 90° joint angle without wing crack or anti-wing crack. The specimens with joint angles of 15°–75° show tensile and shear failure, and the shear failure gradually dominates with the increase of joint angle. The most serious shear failure is observed in specimens with joint angles of 45°–75°, and the wing cracks all start at the joint end, which is obviously different from the specimens of 0° joint angle. The crack initiation angle decreases with the increase of joint angle.

As per the microscopic analysis, the pore structure inside the specimen changes obviously after impact. From the point of view of amplitude growth of signals (as shown in Figure 9 T_2_ distribution), the first peak value of specimens with joint angles of 0°, 15°, and 90° decreases, with less new micropores in specimens and more slightly larger pores. The first peak value of specimens with joints angles of 30°–75° increases, with the number of new micropores increased. Through T_2_ distribution of the specimen, the amplitudes of the second and third peaks have increased significantly, indicating that both medium and large pores have developed obviously under the impact load, with the larger pores being the most prominent. The connection between the three spectral peaks becomes smoother after the impact, indicating that the connectivity between pores of different sizes is better, but there is still an obvious discontinuity between the first and the second peak of the specimens with joints angles of 0°, 15°, and 90°. Under the same impact load, with the increase of joint angle, the number of small pores transformed to medium and large ones in the specimen increases first and then decreases, and the crack development of the specimen is dominated by small pores.

### 3.3. Analysis of Crack Propagation Process

#### 3.3.1. Model Selection and Parameter Determination

The crack propagation process is not obtained in the SHPB test, nor is the change of the stress zone during the failure process of the specimen cannot be obtained. Therefore, LS-DYNA software is used to carry out the numerical simulation of dynamic fracture process of jointed specimens under the same impact pressure. The crack propagation law of the specimen is further supplemented by analyzing the variation of stress isolines. To make better use of simulation software to restore the crack propagation process of jointed specimens and ensure the consistency between the simulated crushing effect and the physical test results, the *HOLMQUIST_JOHNSON_CONCRETE(HJC) is finally selected to simulate the crack propagation process of specimens, according to a large number of studies on the constitutive model of cement mortar specimen under the dynamic load made by experts and scholars [29,30,31,32,33]. The yield surface equation of the HJC constitutive model can be expressed as:

(7) $$\left\{\begin{array}{l} \sigma^{*}=\left[A\left(1-D\right)+BP^{*N}\right]\left(1+C\ln{\dot{\varepsilon }}^{*}\right)\\ \sigma^{*}=\sigma /f_{c}^{\prime }\\ P^{*}=P/f_{c}^{\prime }\\ {\dot{\varepsilon }}^{*}=\dot{\varepsilon }/{\dot{\varepsilon }}_{0} \end{array}\right.$$

where σ* and *σ* are, respectively, the standardized equivalent stress and the actual equivalent stress; *A*, *B*, *C*, and *N* are the correlation characteristic coefficients; P*, *P* are, respectively, the standardized hydrostatic pressure and the actual hydrostatic pressure; *D* is the damage degree of the specimen; *f*_c_′ is the uniaxial compression strength. ${\dot{\varepsilon }}^{*}$, $\dot{\varepsilon }$, and ${\dot{\varepsilon }}_{0}$, respectively, the standardized strain rate, actual strain rate, and reference strain rate.

At present, most of the characteristic coefficients *A*, *B*, *N*, and the maximum standardized equivalent stress σ^*^_max_ used in the HJC constitutive model of cement mortar are mainly specific to the cement mortar specimen with the strength of about 48 MPa, so the above strength parameters need to be adjusted. According to the research results of Zhang et al. [34] on the revision of HJC strength parameters of low-strength concrete materials, it is determined that the bond strength *A* is 0.23, the pressure hardening value *B* is 1.84, the pressure hardening index *N* is 0.88, and the standardized equivalent stress σ^*^ is 7.0. According to research results of Wang et al. [35], the strain rate effect parameter *C* is 0.006. Based on the physical and mechanical parameters of specimen obtained from the uniaxial compression test, this paper calculates the elastic model *E*, shear modulus *G*, bulk modulus *K_e_*, and tensile strength *T*, crushing volumetric strain *μ_c_*, locked volumetric strain *μ_l_*, and others parameters. The results are shown in Table 4, and the relevant parameter calculation Equations (8)–(13) [34,35] is as follows.

The elastic modulus *E* can be calculated, according to the uniaxial compression strength *f*_c_*^’^* and the initial density of cement mortar specimen *ρ*_0_:

(8) $$E=0.043\rho_{0}{}^{3/2}\sqrt{f_{c}{}^{\prime }}$$

The calculation result *E* of Equation (8) and the Poisson’s ratio *v* of the specimen are put into Equations (9) and (10) to obtain the shear modulus *G* and bulk modulus *K_e_*

(9) $$G=E/2\left(1+v\right)$$

(10) $$K_{e}=E/3\left(1-2v\right)$$

The tensile strength *T* can be calculated by Equation (11)

(11) $$T=0.62\sqrt{f_{c}{}^{\prime }}$$

The crushing volumetric strain *μ_c_* and locked volumetric strain *μ_l_* can be calculated by Equations (12) and (13)

(12) $$\mu_{c}=p_{c}/K_{e}$$

(13) $$\mu_{l}=\frac{\rho_{\mathrm{grain}}}{\rho_{0}}-1$$

where *p_c_* is the pressure when the specimen is crushed, and the original parameter of HJC constitutive model is 7 MPa; the density of the specimen after compaction *ρ*_grain_ is 2311 kg/m^3^.

Holmquist et al. assumed that the damage parameters of the specimen had no relation with its strength, so *D*_1_, *D*_2_, and EFMIN adopts the original model parameters: 0.01, 1.0, 0.01.

#### 3.3.2. Model Establishment and Result Analysis

**Figure 10:**
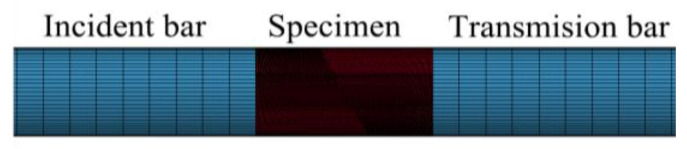
Numerical model of SHPB systems and specimen.

In this simulation, 3D Solid 164 elements are used for modeling, and the model contains incident bars, transmission bars, and specimens with joints of different geometric characteristics. The dimensions of the incident bars and transmission bars are in line with the actual specifications of the SHPB device, both of which are steel cylinders with 1500 mm in length and 50 mm in diameter. The specimen is a cylinder with a length of 100 mm and a diameter of 50 mm. The size and shape of the preset joints are consistent with those of the actual ones. The hexahedral mapping mesh generation is used. With the specimen as the main observation object in the numerical simulation test, the mesh generation number of the incident bars and the transmission bars can be appropriately reduced to improve the calculation efficiency. The number of mesh elements of the incident bars and transmission bars is controlled at about 150,000. The mesh of the specimen can be properly refined, and the number of elements can be about 330,000. The SHPB system and specimen model are shown in Figure 10.

In the simulation, the loading method is realized by inputting half-sine pressure wave on the front-end face of the incident bar. The half-sine wave is converted from the incident wave obtained from the physical experiment, which is closer to the real test. During the calculation process, the *MAT_ADD_EROSION failure criterion is used to control the element failure, and the process of crack propagation is demonstrated by removing the failure elements. This paper selects the stress isogram map and crushing effect of specimens with no joints, 0°, 15°, 60°, and 90° joint angles for analysis and explanation.

The main crack propagation pattern of the non-jointed specimens through the simulation test is consistent with that in the SHPB test results. The tensile crack is formed in the middle of the specimens, with more serious damage at both ends. At the initial loading stage (320 μs), there is no disturbance in the one-dimensional stress wave propagation in specimens. Due to compressive stress and tensile stress, the failure occurs near the high stress area on the two end faces of the specimen. A main tensile crack is developed near the middle of the upper end face of the specimen (the end in contact with the incident bar) to the lower end face of the specimen (the end in contact with the transmission bar), and this main tensile crack continues to develop, confluent with the failure zone on the lower end of the specimen.

For the specimen with 0° joint angle, when the one-dimensional stress wave is loaded up to 320 μs, the stress wave spreads to the preset joint, and the stress concentration begins to appear at the two tips of the joint. Then the principal stress zone with obvious symmetry is formed at the joint, with the stress concentration at the joint tip obviously enhanced. Under further action, the preset joint is compacted, and a new crack appears at the two tips of the joint and the lower left side of the joint. With the continuous action of stress waves, cracks develop on both sides of the joint (A_1_, A_2_, A_3_, A_4_ in the figure), and the principal stress zone moves towards the middle of the specimen with the crack. When the one-dimensional stress wave is loaded up to 450 μs, the two cracks on the upper side of the joint merge into one, so are the two cracks on the lower side. Due to the Poisson’s effect of the specimen, high stress zones (D_1_, D_2_ in the figure) appear along the left and right sides of the weak surface of the specimen, where failure is prone to occur. With further action, the crack is connected with both ends of the specimen, and the crack at the two tips of the joint develops slightly and merges with the tensile crack.

For the specimen with preset 15° joint angle, the stress concentration begins to appear at both ends of the joint at the initial stage of stress wave action. As the stress wave arrives at the upper right end of the joint first, the contour density at the upper right end is slightly higher than that at the lower left end. When the one-dimensional stress wave is loaded up to 350 μs, under the influence of tensile stress, the wing crack initiates at both ends of the joint, and obvious tensile stress zones (A_1_, A_2_ in the figure) appear at the crack tip. Under continuous action, a high stress zone (B_1_, B_2_ in the figure) appears in the opposite direction of the initiation of the wing crack, in which the damage is prone to appear and anti-wing cracks are easy to form. At the same time, there are high stress zones (C_1_, C_2_ in the figure) on the upper right and lower left of the joint tip, in which secondary coplanar cracks are easy to form. When the one-dimensional stress wave reaches 440 μs, the high stress zone (D_1_, D_2_ in the figure) appears on the left and right sides of the specimen due to the Poisson’s effect, and wing cracks, anti-wing cracks, and secondary coplanar cracks all develop. When the wing crack is connected with the upper and lower end faces of the specimen, secondary cracks basically stop developing.

For the specimen with the joint angle of 60°, the stress concentration at both ends of the joint gradually increases at the initial stage of stress wave action, the wing cracks at both ends of the joint initiate, and the stress zones A_1_ and A_2_ at the crack tip move towards both ends of the specimen. When the stress wave is loaded up to 390 μs, the secondary coplanar cracks appear at both ends of the joint, and the stress value is lower than that when the secondary coplanar cracks initiate for the specimen with 15° joint angle, indicating that less stress is required for shear failure of the specimen with 60° joint angle which is more prone to shear failure. The secondary coplanar cracks develop further and are connected with the side of the specimen, the wing cracks connected with the upper and lower end faces. At 490 μs, high stress zones D_1_ and D_2_ appear on the lower left and upper right sides of the specimen, more prone to failure, in good consistence with the SHPB test results.

**Figure 11:**
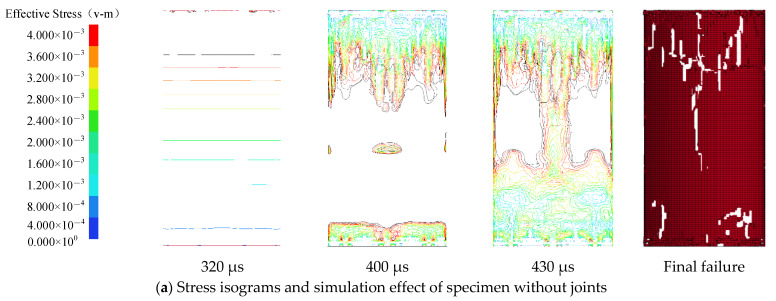

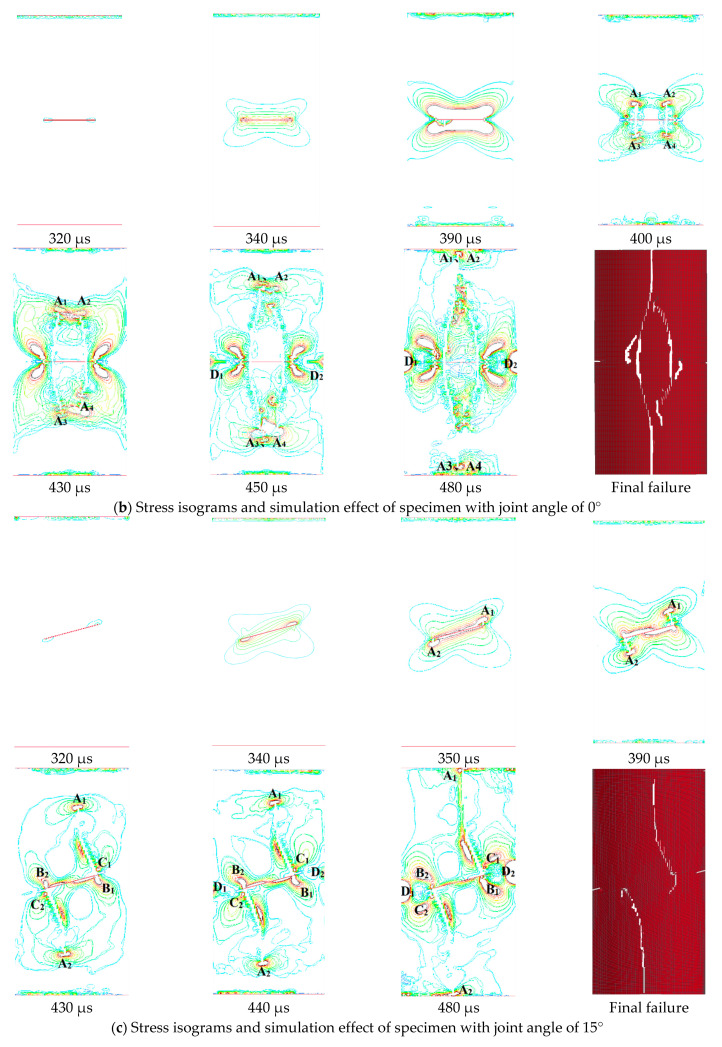

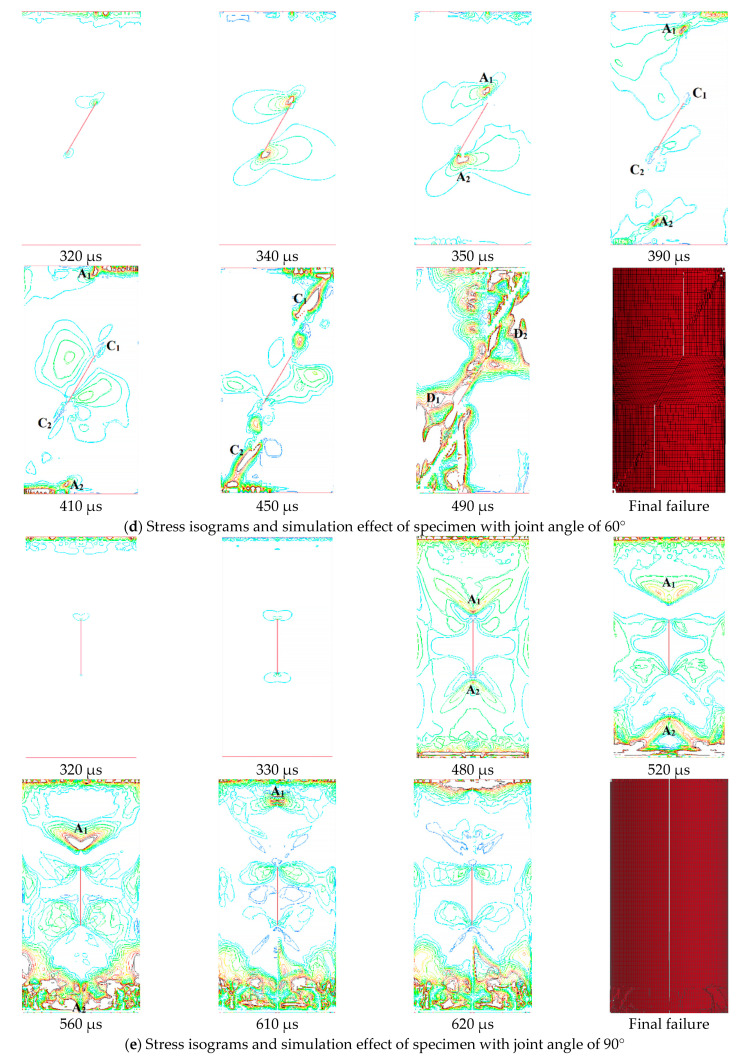
Stress isograms and numerical simulation effect of specimens.

Figure 11e shows the process of stress zone movement and crack propagation of the specimen with the joint angle of 90°. The stress concentration occurs successively at the upper and lower ends of the joint after the action of stress wave. As the tensile stress zone at both ends of the joint moves towards the upper and lower end faces of the specimen, the tensile cracks gradually expand and connect with the upper and lower end faces of the specimen. When the stress wave is loaded up to 520 μs, a large area of failure appears at the bottom of the specimen.

The stress concentration at the joint is regarded as the active force point. Since the specimen will be subject to the relative sliding in the process of failure, the development and propagation of each crack are mutually independent, so there is no symmetry in cracks generated. Compared with the stress wave propagation of specimens without joints, the existence of joints causes obvious stress concentration at the joints of specimens. With the increase of the joint angle, the initial tensile stress zone (A in the figure) gradually transfers to the joint tip, which indicates that the initiation position of the secondary crack gradually moves from the middle of the joint to the two tips of the joint. Meanwhile, the shear stress zone C has an increasingly obvious effect reaching the maximum value at 60°. It is easy to see from the change process of stress isogram with time that the transfer of stress zone at the crack tip is the root cause of secondary crack propagation. From the occurrence order of secondary cracks, the wing cracks always take precedence over the anti-wing cracks and the secondary coplanar cracks.

## 4. Discussion

### 4.1. Relationship Between Joint Angle and Crack Initiation Angle

**Figure 12:**
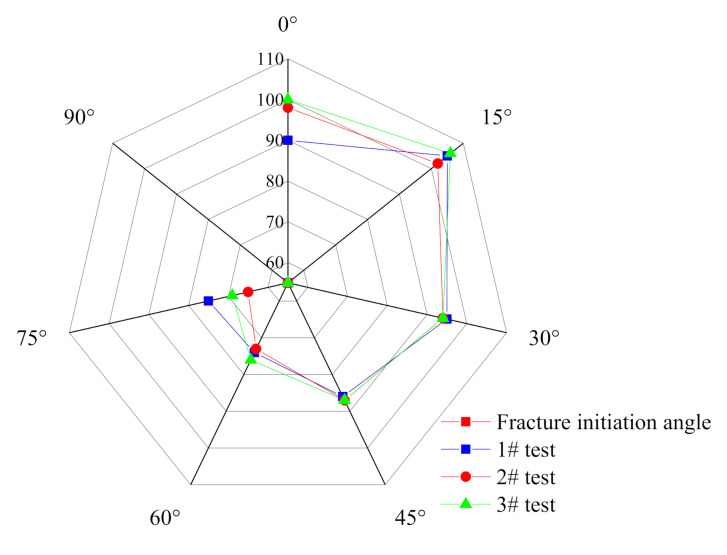
Relationship between joint angle and crack initiation angle.

As shown in Figure 12, as a whole, the crack initiation angle of the specimen gradually decreases with the change of the preset joint angle. Some 0° specimens show splitting failure, so the crack initiation angle is 90°. When specimens are subject to axial action, the joints are compacted, which is also confirmed by the stress–strain curve. At this point, the specimens can be regarded as non-jointed, and the splitting failure is formed due to the Poisson effect. With the increase of the joint angle, the stress point gradually approaches the end of the joint, and the distance of crack propagation decreases. Therefore, the initiation angle decreases gradually. Since all specimens of 90° are with fracture failure and the initiation angle is 0°, the center point is used to replace it for the convenience of distinguishing.

### 4.2. Analysis of Dynamic Penetration Ration

**Figure 13:**
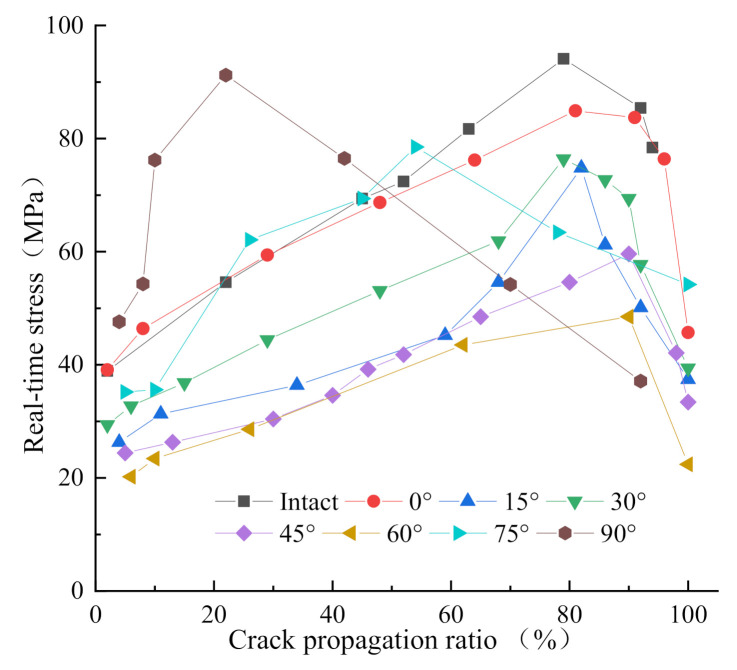
Relation diagram of crack penetration rate and real-time stress.

To better analyze compare the response of joint angle to dynamic load, the penetration rate is defined as the ratio of the visible crack propagation length to the length of the specimen. Through the extraction of numerical model information points, the stress at the end of the specimen is obtained when the crack expands to a certain point. Figure 13 shows the relation between the penetration ratio of the specimen crack relative to the original specimen and the stress at the end of the specimen at the time of crack propagation in the simulation process. It can be seen that after the crack initiation and propagation, the stress borne by the specimen first increases with the crack propagation. According to the analysis of the stress–strain curve in Figure 5, the specimen is now in the stage of energy absorption, and the energy exerted by the stress is applied to the closure, penetration, and propagation of the pores inside the specimen. Therefore, the energy is in the state of accumulation, and the real-time stress increases. The real-time stress, after reaching the peak value in the whole process of crack penetration, begins to decrease with the increase of penetration of ratio. The peak point is the extreme value point of the crack energy storage, after which the crack continues to propagate as a process of energy consumption. It is found that the real-time peak stress point can be reached, and there is a distinct difference in the real time peak points between the specimen with 90° joint angle and that with 75° joint angle when the crack penetration ratio is 80~90%. As can be seen from Figure 9g and Figure 11e, the directions of specimens of 90° and 75° joint angles are the closest to that of crack coalescence, which plays a role in energy guidance. Therefore, the specimens with joint angles of 90° and 75° are the first to reach the peak value in the whole coalesce process. In Figure 9h, the nuclear magnetic resonance scanning shows that the end porosity of the specimen with 90° joint angle increases significantly after the impact, which is higher than that of the non-impacted end, indicating that the direction of action is relatively concentrated after energy passes through the joint.

### 4.3. Analysis of Numerical Simulation Stress and Test Stress

**Figure 14:**
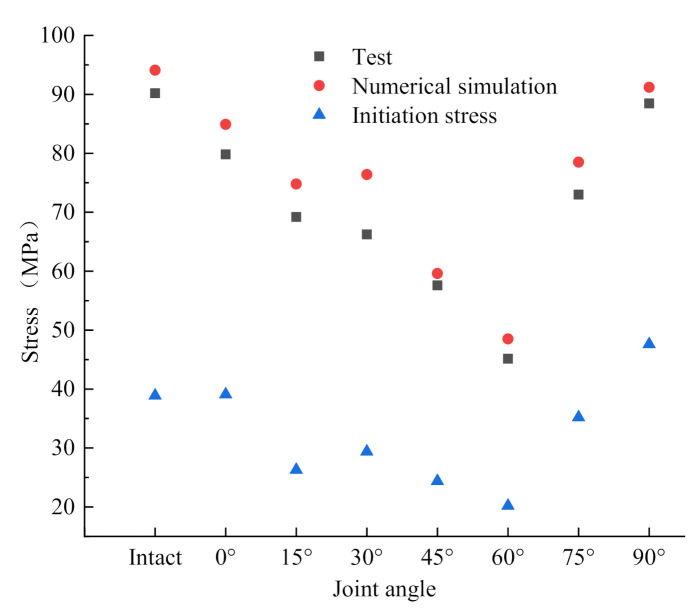
Relation diagram of stress value of numerical simulation and test stress in test.

As shown in Figure 14, specimens with 45° joint angle have the closest two peak values, but the peak stress obtained from the overall simulation results is slightly higher than that in the test results, since the material model selected in the simulation is homogeneous, while the test specimens processed have inevitable pore distribution. However, the overall results are in line with the test results, which jointly verify that the joint angle has a non-negligible influence on the dynamic strength of specimens [36]. When the direction of load effect is 30°~45° from the joint, rock materials containing joints are the most vulnerable to failure. The critical stress of crack initiation in specimens is mostly distributed in the range of 20~40 MPa, which is close to the static compressive strength of specimen. It should be noted that the critical value of crack initiation of the specimen with 90° joint angle is slightly larger, about 45 MPa. Combined with the stress isogram in Figure 11e, the high stress concentrates at the joint end for a long time, so the simulated critical stress is slightly higher than that of other specimens. On the other hand, it can be seen from the study of Mehrdad et al. [37] that more cracks are initiated in the specimens with the preset joint angle of 90° than other angles, so the critical stress is large when the pores are connected and crack is initiated.

Also, it should be noted that due to the lack of confining pressure in the test and simulation, and the relatively complicated number of joint angels in rock masses in engineering practice, the application scope is limited. In future research, more complex working conditions will be studied, such as increased joints, confined pressure constraint forms, changeable preset joint forms, comparative analysis of the influence of conjugate joints and parallel joints on rock strength, varied strain rate, cyclic impact, and dynamic response of the specimen under each joint mode. As research continues, the test equipment will be able to break through the existing limitations to achieve real-time observation of changes in the pores inside specimens during the action. It should be pointed out that, due to limitations of the test conditions, the results of this study can only provide a reference for the dynamic loading area with a strain rate of 10^2^–10^4^ in the project.

## 5. Summary and Conclusions

This paper introduces the preparation and test scheme of specimens with different joint angles and analyzes the rules of influence of joint angles on energy dissipation, crack growth in specimens, and propagation of cement mortar specimens through SHPB impact test, the nuclear magnetic resonance technology, and numerical simulation method, which can be summarized as follows:

(1) The existence of joints influences the energy absorption of specimens. The energy dissipation of specimens can be better analyzed by introducing the energy density per unit time index and combining with the specific energy. When specimens are subject to the external load, the joint angle of 0–30° hinders the propagation of the stress wave. For specimens with 45–60° joint angles, the dissipation energy ratio is at a high level, more prone to instability and failure. The energy ratio of some specimens is relatively discrete, but it does not affect the analysis of the whole law.

(2) The joint form affects the propagation form of the crack, and the existing joint form changes the main direction of energy action, thus affecting the final distribution of the crack in the specimen. However, the crack at the end face of the specimen is roughly located in the same area. Combined with the NMR scanning results, the density of specimen without joints increases uniformly in the circular pores. With prefabricated joint specimens, pores with increased density are concentrated around the cracks of the joint, and the effect of impact energy is more concentrated, so the joint can affect the energy effect.

(3) The whole process of crack initiation, development, and propagation can be analyzed with the help of data simulation, and the reliability of test results can be verified by comparing the simulation results with the test results. The beginning of the variation trend of numerical simulation results is slightly different from that of peak strength measurement, but the overall trend is close. The existence of joints changes the original action distribution of stress and produces an obvious stress concentration at both ends of joints.

## Figures and Tables

**Table 1:** Geomechanical properties of cement mortar specimens.

No.	Density (Kg·m^−3^)		Compressive Strength (MPa)		Elastic Modulus (GPa)		Poisson’s Ratio	
	Actual	Average	Actual	Average	Actual	Average	Actual	Average
B-1	2112.65	2140.11	30.59	31.65	23.89	24.36	0.31	0.30
B-2	2166.12		28.78		22.84		0.33	
B-3	2141.56		35.58		26.34		0.27	

**Table 2:** Specimen grouping scheme.

Test Type	Group	Joint Angle	Impact Pressure (MPa)	Quantity (nos.)
Static test	B	No joint	—	3
Impact pressure determination	A	No joint	0.09 0.11 0.13	3
Impact test without joint	C	No joint	0.11	3
Impact test with joint angle difference	0°	0°	0.11	3
	15°	15°	0.11	3
	30°	30°	0.11	3
	45°	45°	0.11	3
	60°	60°	0.11	3
	75°	75°	0.11	3
	90°	90°	0.11	3

**Table 3:** Energy density per unit time of specimens with different joint angles.

Serial Number	Dissipated Energy *E*_D_ (J)	Specimen Volume *V*_S_ (cm^3^)	Reflected Wave Time *T*_R_ (µs)
0°-1	19.26	165.92	364.1
0°-2	18.45	167.61	339.3
0°-3	21.25	166.93	372.9
15°-1	21.11	165.10	367.6
15°-2	24.42	165.65	339.3
15°-3	22.13	164.57	341.1
30°-1	22.37	166.36	364.1
30°-2	25.60	168.73	307.5
30°-3	21.47	167.36	312.8
45°-1	—	—	—
45°-2	34.66	167.30	341.1
45°-3	26.44	166.28	307.5
60°-1	25.88	166.26	307.5
60°-2	30.97	163.62	341.1
60°-3	28.11	163.68	319.9
75°-1	20.47	165.94	293.4
75°-2	28.77	167.87	293.4
75°-3	20.76	167.49	339.3
90°-1	25.46	167.68	372.9
90°-2	28.70	164.49	323.4
90°-3	20.34	169.49	401.2

**Table 4:** Part parameters of HJC constitutive model of cement mortar.

*E* (GPa)	*G*(GPa)	*T* (MPa)	*μ_c_*	*μ_l_*
24.36	9.37	3.488	0.000345	0.07985

## Data Availability

The datasets generated and analyzed during the current study are available from the corresponding author upon reasonable request.
